# *Plasmodium falciparum* variant erythrocyte surface antigens: a pilot study of antibody acquisition in recurrent natural infections

**DOI:** 10.1186/s12936-017-2097-0

**Published:** 2017-11-07

**Authors:** Elise Schieck, E. Jane Poole, Anja Rippert, Judy Peshu, Philip Sasi, Steffen Borrmann, Peter C. Bull

**Affiliations:** 1 Kenya Medical Research Institute/Wellcome Trust Research Programme, Center for Geographic Medicine Research-Coast, P. O. Box 428, Kilifi, 80108 Kenya; 2 Institute of Hygiene, University of Heidelberg School of Medicine, 69120 Heidelberg, Germany; 3 International Livestock Research Institute, Old Naivasha Road, Nairobi, Kenya; 4 Department of Infectious Diseases, Molecular Virology, University Hospital Heidelberg, Heidelberg, Germany; 5 Department of Clinical Pharmacology, School of Medicine, Muhimbili University of Health and Allied Sciences, P.O. Box 65010, Dar es Salaam, Tanzania; 6 German Center for Infection Research (DZIF), Wilhelmstraße 27, 72074 Tübingen, Germany; 7 Institute for Tropical Medicine, University of Tübingen, Tübingen, Germany; 8 Nuffield Department of Medicine, Centre for Tropical Medicine, Oxford University, Oxford, OX3 7LJ UK; 9 Department of Pathology, Cambridge University, Tennis Court Road, Cambridge, CB2 1QP UK; 10 Present Address: International Livestock Research Institute, Old Naivasha Road, P.O. Box 30709, Nairobi, 00100 Kenya

**Keywords:** *Plasmodium falciparum*, Malaria, Recurrent infection, Recrudescent infection, Antibody aquisition

## Abstract

**Background:**

During intra-erythrocytic replication *Plasmodium falciparum* escapes the human host immune system by switching expression of variant surface antigens (VSA). Piecemeal acquisition of variant specific antibody responses to these antigens as a result of exposure to multiple re-infections has been proposed to play a role in acquisition of naturally acquired immunity.

**Methods:**

Immunofluorescence was used to explore the dynamics of anti-VSA IgG responses generated by children to (i) primary malaria episodes and (ii) recurrent *P. falciparum* infections.

**Results:**

Consistent with previous studies on anti-VSA responses, sera from each child taken at the time of recovery from their respective primary infection tended to recognize their own secondary parasites poorly. Additionally, compared to patients with reinfections by parasites of new merozoite surface protein 2 (MSP2) genotypes, baseline sera sampled from patients with persistent infections (recrudescence) tended to have higher recognition of heterologous parasites. This is consistent with the prediction that anti-VSA IgG responses may play a role in promoting chronic asymptomatic infections.

**Conclusions:**

This pilot study validates the utility of recurrent natural malaria infections as a functional readout for examining the incremental acquisition of immunity to malaria.

**Electronic supplementary material:**

The online version of this article (10.1186/s12936-017-2097-0) contains supplementary material, which is available to authorized users.

## Background

Children who grow up in malaria endemic areas develop naturally acquired immunity to severe forms of the disease. This immunity takes several years to build up, even in hyperendemic areas, and probably never confers sterile immunity. Thus, many individuals in malarious areas carry asymptomatic infections. The transition from malaria to asymptomatic infection is still poorly understood. The staggered age distribution of severe malaria, mild malaria and asymptomatic infection suggests distinctive immunological mechanisms that (i) confer protection against pathogenic features of infection (ii) limit parasite replication leading to control of parasite burden. The role of antibodies is demonstrated through studies in which purified IgG from immune adults was used to bring about rapid reduction in parasitaemia and symptoms of malaria [1, 2]. One novel way to explore this transition further is through observation of recrudescences, which can occur when drug treatment fails to eliminate a blood stage infection [3].

A major target for protective antibodies against malaria are the variant surface antigens (VSA) that are anchored in the cell membrane of infected erythrocytes. These antigens mediate adhesive parasite-host interactions thought to be essential for evading innate immune clearance mechanisms in the spleen. Variant specific anti-VSA immune responses are thought to play an important role in the development of naturally acquired immunity to malaria [4–6]. Following an episode of malaria individuals develop anti-VSA antibodies that are highly specific to the isolate that caused that episode of malaria (henceforth termed homologous parasites) [4–8]. Carriage of anti-VSA antibodies imposes a selection pressure on the infecting blood stage parasite population restricting the diversity of VSAs expressed [4, 5, 9]. More directly an in vivo switch in VSA expression has been observed with accompanying acquisition of antibodies specific to the respective VSAs [10].

*Plasmodium falciparum* Erythrocyte Membrane Protein 1 (PfEMP1), encoded by the large multigene *var* family is thought to be the major VSA [11]. Each haploid genome contains about 60 *var* genes, and expression is mutually exclusive at the individual parasite level (reviewed in [12]). Immune selection of PfEMP1 expression by host antibodies has been shown indirectly by correlation of expression of specific subsets of *var* genes with age [13, 14], and by negative association with anti-VSA antibody repertoire [14–16].

Here, to explore the transition from malaria disease to asymptomatic infection and the relationship between recrudescence and immunity to VSA, anti-VSA antibody acquisition was studied in children within the age bracket where the transition from symptomatic to asymptomatic infections occurs, who presented to hospital with mild malaria, and who returned within 84 days with recurrent parasitaemia. The kinetics of antibody acquisition to the baseline and recurrent isolates was studied. The anti-VSA antibody repertoire of sera from patients that carried parasites that recrudesced was compared to the repertoire of those that experienced reinfection by new genotypes only.

## Methods

### Origin and storage of samples

In this study, samples from children presenting with mild malaria who were included in a Phase III, randomized, non-inferiority trial was used to assess the efficacy and safety of dihydroartemisinin-piperaquine (Artekin) in comparison with artemether–lumefantrine (Coartem©) in Pingilikani, Kenya, with a follow up period of 84 days. This study has been described in detail elsewhere [17]. Children were enrolled if presenting with uncomplicated malaria and fulfilling all criteria as described [17]. They were monitored for 3 days on-site during the administration of therapy and the mother/guardian was asked to return with the child for scheduled visits on days 7, 14, 21, 28, 35, 42 and 84 post-enrollment, or if any symptoms occurred. During this follow up period, samples (serum, blood samples for gDNA extraction and live *P. falciparum* isolates) were collected at any time the children were parasitaemic according to microscopic examination as described [17]. Serum was additionally collected on days 3, 7, 14, 28 and 42. All samples were anonymized. Serum was stored at − 80 °C. Full blood containing parasitized red blood cells was collected and mixed with cryosolution (28% glycerol, 3% (w/v) sorbitol, 0.64% saline) at a volumetric ratio of about 1:4 (packed cell volume:cryosolution) and stored in liquid nitrogen.

### Description of isolates used

During the follow up period recurrent parasitaemias were screened for recrudescences using MSP2 genotyping. Initial screening was done using the RFLP method described by Felger et al. [18] and isolates from 9 patients were selected for the study described here if the patients harboured a recrudescence within the first 42 days of follow up and if the parasitaemia was > 0.1% (Tables 1 and 2). Flow cytometry data was obtained from 7 of these patients, and out of these, 6 patients could later be confirmed as harbouring recrudescence by a superior resolution capillary electrophoresis MSP2 genotyping method [19] using oligo N5 with JOE instead of VIC, on the ABI genetic analyzer 3130 (Additional file 1: Table S1). However, it cannot be excluded that this will include false recrudescences by infections caused by parasites containing the same MSP2 alleles.

**Table 1:** Sample (parasite isolate) characteristics

	Mean ± SD
Age of patient (at inclusion in study)	32.9 months ± 9.9
Time to first recurrence	35 days ± 7.0
Number of MSP2 alleles (baseline infections)	2.29 ± 0.95
Number of MSP2 alleles (recurrent infections)	2.25 ± 2.01
Parasitaemia (baseline infections)	3.3% ± 2.3
Parasitaemia (recurrent infections)	1.4% ± 1.9

**Table 2:** Parasite isolates included in the study

Patients	Female (f)/male (m)	Age (months)	Blood group	Day of parasit-aemia	Parasitaemia (%)	Flow cytometry analysis	*Asymptomatic (tympanic temperature* < *37.5* *°C)*	No of infecting clones^a^
119	F	41	A	0	6.25	Yes		2
				35	0.68	Yes	*No*	2
				84	2.19	No	*No*	1
150	M	30	A	0	1.69	No		3
				42	6.09	Yes	*No*	2
171	M	13	AB	0	2.57	Yes		3
				21	0.07	Yes	*Yes*	1
				76	2.06	Yes	*No*	2
178	M	35	A	0	2.71	Yes		6
				42	4.11	Yes	*No*	1
221	M	38	A	0	6.76	Yes		7
				35	0.27	No	*Yes*	4
				78	0.14	No	*Yes*	7
233	M	42	0	0	2.22	Yes		3
				35	0.47	Yes	*Yes*	7
245	F	31	0	0	1.01	Yes		4
				35	0.11	No	*Yes*	6
				63	0.67	Yes	*Yes*	2
				84	0.11	Yes	*Yes*	9

^a^According to MSP2 analysis (*capillary electrophoresis*)

### Description of sera used

Sera from these 7 patients, taken at day of recruitment and at follow-up visits (days 0, 3, 7, 14, 28, 42 and 84, and when patient visited clinic with parasitaemia during the follow-up period), were used to study antibody acquisition towards the respective infecting parasites in each of these patients over time. Whenever a serum is tested against an isolate from the same patient, they are referred to as homologous serum and isolate.

To test heterologous recognition of the isolates, i.e., testing serum derived from different patients than the parasite isolate tested, a panel of heterologous sera (Table 3) from 14 (blood group A) and 4 (blood group AB) patients were chosen. The panel of 4 sera (blood group AB) was used to test parasites from patient 171, the only patient with blood group AB (Table 2). All sera originated from the same study described above, from children who suffered from recurrent parasitaemias within the follow up period. Four of the sera in the panel originated from patients whose isolates were further characterized in this study (119, 150, 178 and 221), but when the serum-isolate pair (derived from the same patient) were tested this was recorded as homologous testing, not heterologous testing. From all 18 patients of the heterologous serum panel, sera from day 0 (acute) and 14 (convalescent) were tested.

**Table 3:** Heterologous serum panel

Patients	Male (m)/female (f)	Age (months)	Blood group	Parasitemia baseline	Day of recurrence	*Recurrent infection asymptomatic (tympanic temperature* < *37.5* *°C)*	Recrudescent infections^a^	Number of infecting clones^a^	
								Baseline infections	Recurrent infections (recrudescent/new infections)^a^
56	M	44	AB	0.10	49	*Yes*		1	0/1
77	M	34	A	0.08	35	*No*	Yes	5	3/3
105	M	43	AB	1.29	49	*Yes*		1	0/1
111	M	36	A	0.11	28	*Yes*		1	0/1
115			AB	1.5	49	*Yes*		1	0/3
119	F	41	A	6.25	35	*No*	Yes	2	1/1
127	M	46	A	0.85	45	*Yes*		5	0/1
144	M	48	A	5.05	40	*No*		2	0/1
150	M	30	A	1.69	42	*No*		3	0/2
160	F	10	A	0.56	42	*No*		1	0/4
162	F	25	A	0.06	49	*No*	Yes	2	1/1
169	M	33	A	5.38	42	*Yes*		2	0/2
178	M	35	A	2.71	42	*No*	Yes	6	1/0
181	F	42	A	0.05	42	*Yes*	Yes	4	4/0
191	F	44	A	3.99	54	*Yes*		1	0/2
221	M	38	A	6.75	35	*Yes*	Yes	7	2/2
246	M	40	A	0.27	40	*No*		1	0/10
247	M	32	AB	0.47	56	*No*		5	0/4

Sera from day 0 (acute) and day 14 (convalescent) was used from all patients

^a^According to MSP2 analysis (*capillary electrophoresis*)

Improved MSP2 analysis using capillary electrophoresis [19] was performed as described above on the isolates derived from the patients whose sera were included in the heterologous serum panel to differentiate between sera derived from patients who experienced recrudescences and reinfections during the follow up period.

### Flow cytometry

Live isolates were removed from liquid nitrogen, washed with ice cold 3.5% NaCl, then washed with culture medium (RPMI 1640; Gibco), and transferred into culture flasks where the isolates were allowed to stabilize for 18–24 h under standard culture conditions [20] until trophozoite stage and were then analysed using flow cytometry. The procedure of freezing and thawing of the already small blood sample volumes originating from young children reduces the volume further mainly due to red blood cell lysis. For each test, 0.5 µl of the isolate pellet were incubated 30 min in 40 µl of PBS (0.1% BSA) with 0.1 mg/ml Ethidium Bromide (Sigma) and 1 µl serum. Negative controls were no serum and non-immune pooled blood group AB serum from non-exposed European adults, and a positive control, hyperimmune blood group AB adult serum from the Kilifi area also used in previous studies [14, 21] was included. After washing (with PBS, centrifuging at 900*g* for 5 min) cells were incubated in 30 µl of PBS (0.1% BSA) containing antihuman-CD45-PC7 (1:30 v/v) (IM3548; Beckman Coulter), antihuman IgG-FITC (1:1 v/v) (FA004; the Binding Site) and antihuman IgM-SPRD (1:30 v/v) (735986; Beckman Coulter). After washing three times (with PBS, centrifuging at 900*g* for 5 min) samples were resuspended in PBS (0.1% BSA) and analysed using an FC500 Flow Cytometer (Beckman Coulter).

FlowJo and Excel were used for the initial flow cytometry analysis, where first nucleus containing cells stained by ethidium bromide were included, i.e. leukocytes and infected red blood cells (irbcs) at the trophozoite stage. At this stage ring stage parasites were excluded by low ethidium bromide staining. Next CD45-containing leukocytes were excluded and finally the IgG recognition measured as mean fluorescence intensity (MFI).

The flow cytometer was set to count 1000 irbcs. Samples where less than 200 irbcs (due to small sample volumes and low parasitaemia) had been measured were excluded from analysis. When the volume of cells was smaller than expected (pipetting errors on semi-packed cell pellets), the last cells measured would give unusually many counts with high MFI values. To exclude these erroneous measurements, samples where the whole population of measured cells (mostly noninfected red blood cells) had more than 2.5% IgG positive cells, defined by a cut-off at MFI arbitrary units of 12, were excluded from analysis. Manual analysis confirmed that no samples with high parasitaemia were excluded by this process. All flow cytometry results are presented in this study as the MFI for each isolate-serum pair, with the individual “no serum” sample MFI subtracted. The “no serum” value for each parasite isolate was subtracted rather than the value of the negative control pool to allow comparison of the data with the negative control group. The MFI values for each measurement together with parasite isolate and serum information is presented in the Additional file 1: Table S1.

If the no serum sample was missing (excluded by reasons explained above) or differing by more than 90% from the median of all no serum samples, the whole series, i.e. all data from the corresponding isolate, was excluded.

To ensure that the measured fluorescence was not simply an effect of higher parasitaemia in the sample, i.e. an experimental artefact, linear regression analysis was performed for fluorescence vs. parasitaemia but no correlation was found (P = 0.27, not shown).

All flow cytometry results used for analysis is presented in Additional file 1: Table S1.

### Statistical analysis

Graphpad Prism 6.0e for MAC OS X and Genstat^®^ 16th Edition were used for analysis and graphical presentation. A mixed-effects linear regression model using a REstricted Maximum Likelihood (REML) [22] approach was used in each analysis described below. In all analyses below, random effects for patient and serum were used to allow for correlation in reactivity of sera and isolated parasites originating from the same patient.

To test for intrinsic differences in the ability of the parasites to be recognized, the data were initially transformed by natural logarithm, log_e_ (MFI + 0.145) to make the smallest observation positive and to normalize the distribution. Fixed effects for response type (originating from a patient that responded to its parasite isolate vs. a patient that did not) and serum day (day 0 or 14), together with their interaction were then tested in the model.

The same analysis was performed to test for a difference between recognition of baseline vs. recurrent isolates. In this case the fixed effects were isolate type (baseline or recurrent) and serum day (day 0 or 14).

The ability of homologous sera taken before recurrence to recognize the recurrent parasites was also compared to that of their overall recognition by heterologous sera with a linear mixed effect regression model. In this analysis, only sera taken before the recurrent infection were used. The data were transformed by log_e_ (MFI + 0.062) to make the smallest observation positive. Fixed effects tested in the model were serum type (homologous recognition of recurrent isolates vs. recognition of the same recurrent isolates by heterologous sera) and serum day (day 0 or 14).

To compare the anti-VSA antibody repertoire of children who eliminated the baseline parasites to that of those children who did not, a similar linear mixed effect regression model was used. The data were transformed by log_e_ (MFI + 0.145) to make the smallest observation positive. Fixed effects for serum type (originating from a patient that eliminated its parasite infection vs. a patient that did not) and serum day (day 0 or 14) were fitted.

Histograms or residuals, fitted-value plots and normal plots were performed to test the suitability of the transformed data in the linear mixed effect regression models.

## Results

### Characterization of parasite isolates

For this study, samples from children presenting with mild malaria who were included in a dihydroartemisinin-piperaquine vs. artemether–lumefantrine drug trial were used [17]. In total 14 parasite isolates from 7 of these patients were analysed for antibody recognition (Tables 1, 2). All the children involved are within the age bracket where *Plasmodium* infections transit from causing disease to being asymptomatic. Table 2 also shows whether the recurrent infections were asymptomatic (tympanic temperature < 37.5) at the time of detection.

### Characterization of sera

Sera derived from children in the same study were used to test antibody recognition of the isolates described above. Homologous sera (sera derived from the same patient as the parasite isolate tested) were used to test acquisition of antibodies to the infecting isolates of the respective patients. Sera from all available time points during the study follow up were used.

Heterologous sera (sera derived from other patients than the tested isolate) were used to test general recognition of the isolates in the population. For this a panel of sera from in total 18 patients in the same study were chosen to characterize heterologous recognition of the parasite isolates (Table 3). Sera from day 0 (acute) and 14 (convalescent) were used. All the patients contributing serum to this panel had recurrent infections during the follow-up period of 84 days, and Table 3 shows the day of recurrence and whether the detected recurrent infection was asymptomatic at the time of detection. Active weekly follow-up as in this study is likely to detect an increased number of asymptomatic infections, here > 50% of the recurrent infections were asymptomatic.

### Antibody acquisition to infecting isolates varies between patients

**Fig. 1:**
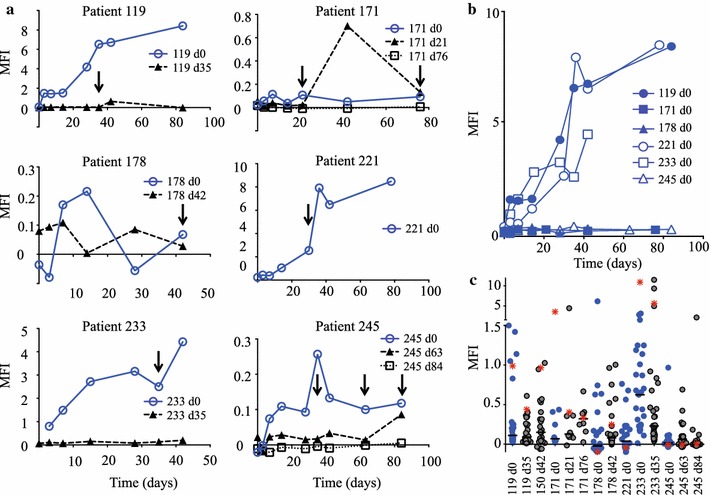
Kinetics of antibody acquisition to baseline and recurrent isolates. **a** Surface recognition of individual parasite isolates by their corresponding homologous sera. Sera taken at different time-points after uptake of the child in the study were tested for recognition of the baseline and recurrent isolates, measured as mean fluorescence intensity (MFI). The recognition of baseline isolates is depicted in blue and that of recurrences in black. Arrows point to the time when the investigated recurrent parasitaemia was detected. **b** Homologous recognition of all baseline isolates is summarized to better illustrate the separation of responders versus non-responders. **c** Heterologous recognition of the individual isolates showing that isolates are variably recognized by heterologous sera, the median is shown as a horizontal bar. Hyperimmune serum recognition of the individual isolates is also shown (red asterisk)

Antibodies to the infected red blood cell (irbc) surface were measured by immunofluorescence and flow cytometry. To explore the kinetics of antibody responses to irbc sampled at baseline (day 0) and at the time of recurrent parasite infections each parasite isolate was tested against homologous sera (sera derived from the same patient as the parasite isolate tested) sampled at different time points. Figure 1a, b shows the kinetics of antibody acquisition. 3/6 patients developed antibodies to their baseline parasites (blue lines), with antibody acquisition rising over time (patients 119, 221 and 233). The other three made either a very weak, or no response at all i.e. patients 171, 178 and 245. This variation in antibody responses appeared not to be due to intrinsic differences in the ability of the parasites to be recognized by antibodies because there was no significant correlation between the homologous and heterologous recognition of these parasite isolates (P = 0.221, mixed-effects linear regression model) (Fig. 1c and Additional file 2: Figure S1), and there was no overall difference in antibodies to baseline vs. recurrent isolates measured in heterologous sera (P = 0.215, mixed-effects linear regression model). One explanation could be the duration of the infection (or more precisely: the infection with the clone or clones that were responsible for the malaria episode) before the patients presented to us. If diagnosed quickly and combined with the fast acting ACT used in this trial parasites could have been removed before an effective antibody response was mounted. This hypothesis could not be tested here.

### Recurrent *Plasmodium falciparum* infections tend to be poorly recognized by pre-existing homologous antibodies

**Fig. 2:**
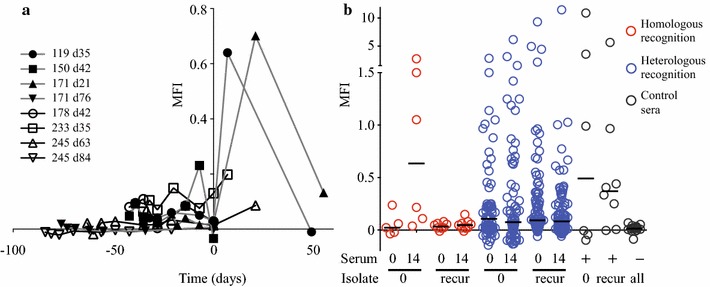
Recurrent infections exploit gaps in antibody repertoire against surface of infected red blood cells. **a** Homologous surface recognition of individual recurrent isolates, where the day of each recurrent parasitaemia is set at day 0. **b** Homologous recognition of all isolates as well as heterologous recognition is shown. Homologous recognition of the isolates is shown in red, and heterologous recognition of the same isolates is shown in blue. For comparison, recognition of all the isolates by hyperimmune and nonimmune sera is also shown, in gray. The sera used was taken at day 0 (at the uptake of the patient in the study), or at day 14 (convalescent sera), as indicated. The isolates were divided into baseline and recurrent isolates, as indicated below the graph. Homologous sera taken before recurrence recognizes the recurrent parasites less than their overall recognition by heterologous sera (P = 0.063). MFI: Mean fluorescence intensity

As shown in Fig. 1a recognition of recurrent parasites by homologous sera taken earlier than the recurrence was generally poor. Figure 2a illustrates this further, where homologous recognition of the individual recurrent isolates is shown and the day of recurrent parasitaemia is set at day 0. To explore this further, this pre-existing homologous recognition of recurrent isolates was compared to recognition by a panel of heterologous day 0 (acute) and day 14 (convalescent) sera from other patients in the same study (Table 3). Recurrent parasites tended to be recognized less well by homologous sera than heterologous sera, although with only borderline significance (Fig. 2b) P = 0.063, mixed-effects linear regression model). Overall these observations are consistent with the idea that parasites express antigens corresponding to gaps in the repertoire of antibodies carried by the host.

### Genotyping

All sera used to test heterologous recognition of the isolates described above were derived from patients who had also experienced a recurrent, i.e. secondary infection during follow-up. Those patients who were unable to clear their parasites in spite of treatment, resulting in a recrudescent infection are of specific interest. Therefore, MSP2 genotyping analysis was performed on the parasite DNA isolated from these patients to differentiate between recrudescence (persistent blood stage infection) and re-infection (new infection emerging from the liver). A recrudescent infection is defined by the presence of at least one genotype during follow-up also detected before treatment. Thus, 6/18 patients whose sera were part of the heterologous serum panel were confirmed to have recrudescent infections within the follow-up period (Table 3).

### Patients carrying recrudescent infections tend to have a broader antibody repertoire than patients who cleared their infections

**Fig. 3:**
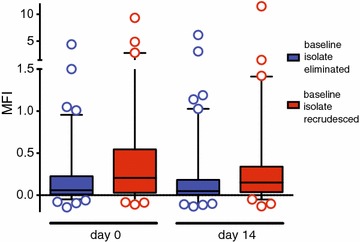
Patients harbouring primary isolates that subsequently recrudesced have higher background recognition than patients that eliminated primary infections (P = 0.059). Shown here is the mean fluorescence intensity (MFI) of the heterologous recognition (of 14 isolates) by sera from 12 patients who eliminated their infections (and were later reinfected) (blue) compared to the 6 patients with recrudescences (red). The results are further divided into sera from day 0 (acute) and day 14 (convalescence) to show that the measured immune response is relatively stable, i.e. does not fluctuate from day to day, and is not due to submicroscopic infections still present in children with recrudescences in day 14 sera. Boxes show the interquartile range, whiskers the 5th–95th percentile and the median is shown as a horizontal bar. Single measurements outside the 5th–95th percentile are shown individually

Next, the anti-VSA antibody repertoires of the 6 patients with recrudescence was compared to the 12 patients who cleared their initial infections (Table 3). For this their ability to recognize the 14 parasite isolates from the 7 patients described above was measured (Table 2 and Additional file 2: Figure S1). The anti-VSA antibody repertoire of the sera from children who carried recrudescent infections was higher than that of children with only reinfections (Fig. 3), although only borderline significant P = 0.059 as tested using a linear mixed effect regression model. To exclude that this is not simply an effect of reduced drug sensitivity of the parasite, existing data from all children in the initial study experiencing recurrences [17] was used to re-analyse risk of recrudescence as a function of the IC_50_ values for the long-acting drugs lumefantrine and piperaquine. A recrudescence is not determined by the sensitivity of the baseline isolate to the long-acting drugs used in the study (Additional file 3: Table S2). There was no indication of tolerance or resistance in the response to dihydroartemisinin in the study [17].

It is possible that submicroscopic infections (undetected microscopically but persisting until the recrudescence was detected), may themselves boost antibodies. However, the anti-VSA antibody repertoire in children who later had recrudescent parasites was higher than in those who later had recurrent infections at day 0 (Fig. 3). This suggests that sustained submicroscopic infection in children with recrudescences was not responsible for the difference in antibody levels between these two groups of children. One explanation for the difference is that the ability to recognize heterologous parasites promotes chronic asymptomatic infection as previously proposed by others [9, 23–25].

## Discussion

Antibodies that bind parasite-encoded antigens on the surface of infected red blood cells (variant surface antigens, VSA) have been implicated in alleviating specific pathogenic interactions between parasite ligands and endothelial host receptors [26], but far less is known about the role of anti-VSA antibodies in benefiting parasite survival. From an evolutionary perspective, it is expected that natural selection favours parasite immune evasion strategies, including the display of VSAs, that increase survival in the form of asymptomatic maintenance of transmission reservoirs.

The transition from *P. falciparum* infections that trigger disease to asymptomatic infections is well documented in populations with high re-infection rates, but the exact nature of the host-parasite interactions that orchestrate this survival strategy of the parasite has been difficult to study, and recurrent *P. falciparum* infections in paediatric patients within an age bracket that encompasses the switch from symptomatic to asymptomatic infections could provide a useful readout.

Children who received treatment for malaria were recruited and were then followed-up to detect recurrence of infections. Stratification of recurrent infections in recrudescent primary infections or new secondary infections gave us a functional in vivo readout to assess the effects of anti-VSA responses determined at baseline.

The pattern of antibody responses relating to malaria and recurrent infections were consistent with previous studies showing that episodes of disease are associated with specific gaps in the anti-VSA repertoire [14–16]. There was a tendency for antibodies in heterologous sera to recognize recurrent isolates to a higher degree than antibodies in day 14 sera recognized parasites from subsequent homologous recurrent isolates.

Persistent primary infections were detected as mild or asymptomatic recrudescences in patients with higher than average heterologous recognition capacity measured at day 0. This is consistent with a potential role of anti-VSA antibodies maintaining chronic infections [9, 23–25]. Recker et al. have proposed a model in which a higher (transient) cross-reactive immune response can potentially slow down the development of long-lived variant specific responses, thereby prolonging the duration of infection [23–25].

A model in which anti-VSA antibodies help sustain chronic infections helps to explain previous observations of higher than average risk of recrudescent infections in young children [3, 27]. This phenomenon in early childhood may be best explained by the immaturity of acquired immune mechanisms that directly contribute to the elimination of infections, and that maturity of these effector mechanisms may be a prerequisite for developing tolerance to subpatient, persistent infections.

This pilot study is limited by a small sample size. Recrudescences are rarely observed after highly efficacious artemisinin-combination therapy, and the definition of a recrudescent infection was based on a repeat polymorphism of a single, if highly variable, gene (MSP2). This method is reliable at a population level, but has limitations at the individual level because there will always be a bias toward majority genotypes. Studies in children also constrain the amount of sample volume, here only one measurement per isolate-serum pair tested was performed. Future larger studies will be required to confirm the hypotheses set out in this study.

It would be very interesting to make a detailed comparison of expressed VSA from recrudescent parasites in comparison with those that are isolated at day 0. It was not possible to do this accurately in this study because blood for which white blood cells had not been removed was used. Standard conditions for DBL-alpha tag amplification cannot be used in the presence of high levels of human RNA. A preliminary analysis using amplification at higher temperature was performed and no major changes in the profile of *var* genes expressed at different time points was observed. Specific studies to analyse such differences in transcriptome between isogenic parasites at different points during the establishment of chronic infections would be of great value in understanding of how these infections become established.

## Conclusions

In short, here data is presented that support the “hole in the wall” model, i.e. a framework for explaining how the parasite exploits gaps in the anti-VSA repertoire of the host. It is suggested that this model may be key to understanding how chronic infections are established in humans. First data is provided to support the hypothesis that a more complete anti-VSA antibody repertoire acquired after repeated infections could favour the transition from an uncomplicated malaria episode to an asymptomatic carrier status. These findings must be confirmed in larger studies, but it opens up to new interpretations of immunological data, and to the usage of recrudescence as an alternative, possibly more useful resource to measure immunity to malaria.

## Additional files

**Additional file 1: Table S1.** Samples and flow cytometry data used for analysis in this article.

**Additional file 2: Figure S1.** Heat map showing heterologous recognition of each individual isolate-serum pair (and homologous recognition highlighted by bold boxes). Mean fluorescence intensity is shown for each isolate-serum pair, and is colour graded in a scale from light green (lowest values), to dark green (highest values).

**Additional file 3: Table S2.** A recrudescence is not determined by the drug sensitivity of the baseline isolate.

## Abbreviations

VSA: variant surface antigens
PfEMP1: *Plasmodium falciparum* Erythrocyte Membrane Protein 1
MSP2: merozoite surface protein 2
irbc: infected red blood cell
MFI: mean fluorescence intensity
