# Supplementary material for: Plasmodium falciparum variant erythrocyte surface antigens: a pilot study of antibody acquisition in recurrent natural infections
Source: Malar J. 2017 Nov 7;16:450. doi: 10.1186/s12936-017-2097-0 (PMC5678811; doi:10.1186/s12936-017-2097-0)
Supplement: Supplementary file 2 — Additional file 2: Figure S1. Heat map showing heterologous recognition of each individual isolate-serum pair (and homologous recognition highlighted by bold boxes). Mean fluorescence intensity is shown for each isolate-serum pair, and is colour graded in a scale from light green (lowest values), to dark green (highest values). [file 12936_2017_2097_MOESM2_ESM.pdf]

Serum (Ptn no)	77	77	111	111	119	119	127	127	144	144	150	150	160	160	162	162	169	169	178	178	181	181	191	191	221	221	246	246	hyperimmune	nonimmune
Day_serum	0	14	0	14	0	14	0	14	0	14	0	14	0	14	0	14	0	14	0	14	0	14	0	14	0	14	0	14	cntrl	cntrl
isolate																														
119 d0	0.25	0.25	1.05	1.14	**0.06**	**1.50**	0.16	0.10	1.50	0.83	0.03	0.05	0.04	0.03	0.21	0.28	0.07	0.06	0.03	0.03	0.20	1.42	0.02	0.09	0.12	0.20	0.04	0.05	0.99	0.05
119 d35	0.36	0.40	0.35	0.61	**0.02**	**0.06**	0.08	0.05	0.25	0.34	0.02	0.05	0.03	0.02	0.13	0.18	0.06	0.02	0.03	0.10	0.24	0.26	0.03	0.05	0.34	0.14	0.02	0.01	0.44	0.02
150 d42	0.31	0.32		1.03	0.29	0.03	0.09	-0.03	0.95	0.41	**0.05**	**0.02**	0.03	0.05	0.53	0.57	0.07	-0.06	0.04	0.15	0.55	0.22	0.01	0.04	0.18	0.15	-0.06	0.15	0.97	-0.01
178 d0	-0.01	0.64	-0.14	-0.12	-0.05	-0.13	0.40	-0.09		0.03	-0.09	6.15	0.01	-0.10		0.11	-0.03	0.15	**-0.04**	**0.22**	0.74	0.63	-0.08	-0.07	0.17	0.19	-0.05	-0.13	-0.10	-0.09
178 d42	0.11	-0.01	0.36	0.01	0.05	0.07	0.02	0.13	0.75	0.09	0.06	0.02	0.05	0.12	0.52	0.57	0.07	0.02	**0.08**	**0.00**	0.96	1.00	0.04	0.07	0.13	0.23	0.09	0.02	0.25	0.02
221 d0	-0.11	0.23	0.56	0.68	0.22	-0.10	0.11	0.06	0.22	0.19	0.16	-0.05	0.00	0.01	0.23	0.76	0.23	-0.10	-0.05	-0.05	0.12	-0.05	-0.03	-0.10	**0.24**	**1.05**	0.00	-0.11	-0.04	0.02
233 d0	1.14		0.23	1.19	0.22	0.31	0.44	0.70	1.01	3.11	0.11	0.87	0.18	0.16	0.63	1.52	0.23	0.01	0.68	1.25	2.80	1.25	0.25	0.18	0.88	0.66	0.45	0.36	10.92	0.07
233 d35	9.33	11.48	0.45	0.24	0.23	0.09	0.17	0.15	0.06	0.42	0.21	0.34	0.10	0.04	0.46	0.12	0.84	0.06	0.68	0.77	4.93	0.35	0.05	0.07	0.92	0.23	0.22	0.14	5.64	0.02
245 d0	0.02	0.04	0.00	0.03	0.01	-0.02	0.00	-0.01	0.00	0.03	-0.01	0.02	0.01	0.01	0.12	0.11	0.01	-0.01	0.04	0.06	0.97	0.09	0.02	-0.03	0.07	0.07	-0.04	0.01	-0.01	0.01
245 d63	0.10	0.25	0.42	0.08	0.08	-0.02	0.07	-0.04	0.07	0.09	0.02	-0.03	0.02	0.00	0.10	0.05	0.00	-0.02	0.05	0.09	0.69	0.06	0.04	-0.03	0.27	0.26	0.06	0.01	0.00	0.03
245 d84	0.05	-0.01		-0.02	0.12	-0.02	0.00	-0.02	0.18	0.01	-0.01	-0.01	0.00	0.00	0.02	0.04	0.00	0.01	0.00	0.01	2.12	-0.01	0.00	-0.02	0.01	-0.01	0.00	-0.02	0.01	-0.01

Serum (Ptn no)	56	56	105	105	115	115	247	247	hyperimmune	nonimmune
Day_serum	0	14	0	14	0	14	0	14	cntrl	cntrl
isolate										
171 d0	0.04	0.44	0.04	0.09	0.02	0.05	0.25	0.41	3.54	0.01
171 d21	0.36	0.36	-0.02	0.09	0.08	0.15	4.41	0.11	0.41	0.04
171 d76	0.16	0.41	0.26	0.38	0.03	0.67	0.25	0.39	0.33	0.00

Figure S1. Heat map showing heterologous recognition of each individual isolate-serum pair (with homologous recognition in bold boxes). Mean Fluorescence Intensity is shown for each isolate-serum pair, and is colour graded in a scale from light green (lowest values), to dark green (highest values).
